# A randomized controlled clinical trial study protocol of Liuwei Dihuang pills in the adjuvant treatment of diabetic kidney disease

**DOI:** 10.1097/MD.0000000000021137

**Published:** 2020-07-31

**Authors:** Tingting Liao, Keni Zhao, Qun Huang, Shiyun Tang, Keling Chen, Chunguang Xie, Chuantao Zhang, Wenfan Gan

**Affiliations:** a Department of Endocrinology; b Department of Respiratory Medicine; c Department of Ophthalmology, Hospital of Chengdu University of Traditional Chinese Medicine, Chengdu, China.

**Keywords:** Chinese herbal medicine, diabetic kidney disease, Liuwei Dihuang pills, randomized controlled trial

## Abstract

**Introduction:**

A large number of patients with diabetic kidney disease (DKD) approach traditional Chinese medicine (TCM) owing to discontent with standard treatments. Based on TCM theory and clinical experience, the syndrome of kidney yin deficiency is a common type of DKD. Liuwei Dihuang pills (LDPs) is a common prescription of a Chinese herbal formula for patients presenting this syndrome of DKD. However, well-established data supporting the efficacy and safety of LDP in DKD treatment are lacking.

**Methods:**

We have designed a double-blind, placebo-controlled, randomized trial. After a 2-week run-in period, 124 eligible participants with DKD will be assigned to either the experimental or the control group in a 1:1 ratio. Patients in the experimental group will receive LDP, while patients in the control group will receive a matched placebo. As the basic treatment in the 2 groups, metformin hydrochloride sustained-release tablets, for blood glucose control, and irbesartan tablets, for blood pressure regulation, will be provided. All participants will undergo 4 weeks of treatment and 12 weeks of follow-up. The primary outcome is the change in 24 hours urinary protein levels, measured from the baseline to the end of the treatment phase (week 24). The secondary outcomes to be assessed include the change in serum creatinine and estimated glomerular filtration rate, urinary albumin excretion rate, improvement of TCM syndromes and symptoms, fasting blood glucose and postprandial 2-hour blood glucose, blood lipids (total cholesterol, triglyceride, high-density lipoprotein cholesterol, and low-density lipoprotein cholesterol, from baseline to weeks 12 and 24.

**Discussion:**

The results of this study will provide high-quality evidence of the effects of LDP in DKD treatment, which will provide an alternative treatment strategy in patients with DKD.

## Abstract 2

Supplemental Digital Content is available in the text

## 1 Introduction

Diabetes is a metabolic disease characterized by hyperglycemia. In 2018, the International Diabetes Federation (IDF) published the 8th edition of the Global Survey of Diabetes, stating that there were 114.4 million people with diabetes between the ages of 20 and 79 years in China, ranking first among all the IDF regions in 2017.^[1]^ Diabetic kidney disease (DKD) is a common microvascular complication of diabetes and is characterized by a clinical syndrome, including persistent albuminuria, elevated blood pressure, a continuous decrease in the glomerular filtration rate (GFR), and increased cardiovascular events and mortality.^[2]^ According to the UK Prospective Diabetes Study, approximately one in every 5 people would develop DKD.^[3]^ However, current statistics regarding the incidence of diabetes in China is lacking.^[4]^ In 2002, statistical data on the incidence of DKD reported that 33.6% of patients with diabetes were diagnosed with DKD in the 10 years from 1990 to 2000, with 22.5% presenting type 1 diabetes and 34.7% presenting type 2 diabetes.^[5]^ With the growing incidence of diabetes, the incidence of DKD has increased and has become the main cause of end-stage renal disease.^[6,7]^ In western medicine, DKD is treated by controlling the blood sugar, blood pressure, blood lipids, and reducing urine protein, with no effective methods to prevent the progression of DKD. Therefore, finding appropriate remedies for the prevention and treatment of DKD, as well as reducing medical costs, are important tasks for resolving this disease.

In the ancient books of traditional Chinese medicine (TCM), there is no record of the term “DKD.” Based on the clinical symptoms, etiology, and pathogenesis of DKD, the disease can be summarized as “edema,” “turbid urine” (murky urine like rice water or grease), “wasting-thirst” (any diseased state characterized by polydipsia, polyphagia, and polyuria, similar to diabetes), and “consumptive disease” (a general term for chronic deficiency diseases due to consumption of yin, yang, qi, and blood) in TCM. Practitioners of modern medicine believe that the etiology, pathogenesis, and clinical symptoms of DKD are markedly similar to those observed in nephropathy and wasting-thirst in TCM. DKD is mainly located in the spleen and kidney, providing the TCM disease name “nephropathy of wasting-thirst.”^[8]^ In the early stage, DKD is characterized by yin deficiency, and the disease is located in the liver, spleen, and kidney. During the middle stage, DKD is characterized by yang deficiency, located in the liver, spleen, and kidney. The end-stage of this disease is characterized by yin and yang deficiency, located in the spleen and kidney. With the gradual aggravation of DKD, the symptoms and syndrome elements of phlegm and blood stasis are aggravated.^[9,10]^

Currently, several experiments have confirmed that TCM prescriptions are effective in the adjuvant treatment of DKD. Zhou^[11]^ and Ai^[12]^ have shown that Qiwei granules are effective in the treatment of stages III and IV DKD with qi and yin deficiency syndrome, as well as the blood stasis syndrome. In animal experimental studies, Qiwei granules can delay the development of DKD by reducing podocyte injury and protecting renal function by upregulating the expression of nephrin^[13,14]^ and CD2AP^[15]^ in renal podocytes of DKD. Tang and colleagues^[16]^ have observed that the Huopu Xialing decoction can protect the kidney of DKD rats by inhibiting the expression of the transforming growth factor-beta 1 (TGF-β1) signaling pathway and increasing the expression of nephrin and podocin in podocytes, thus reducing podocyte apoptosis. Zhang and Yue^[17]^ have reported that the Jiangtang Baoshen decoction improves clinical outcomes in DKD patients by regulating blood lipid, blood glucose, and the urinary protein excretion rate in early type 2 DKD patients. Lu^[18]^ has documented that the Jiawei Shenling Baizhu powder granules can protect the kidneys of early DKD patients presenting spleen and kidney qi deficiency and blood stasis. Recent studies have confirmed that TGF-β1 plays an important role in the development of DKD. Liu et al^[19]^ has observed that the Compound Coptis Capsules improve the pathological changes occurring in renal function during early DKD in rats, delaying the chronic pathological progression of DKD. Here, the mechanism may be related to the regulation of the imbalance of TGF-β1/BMP-7 expression in DKD through the Smad signaling pathway. Li et al^[20]^ have documented that the Tongyu decoction combined with *Tripterygium* glycosides relieves the clinical symptoms and effectively reduces blood glucose levels. Additionally, it can regulate the levels of TGF-β1, platelet-derived growth factor-BB, and connective tissue growth factor, and improves renal function and clinical efficacy.

Liuwei Dihuang pill (LDP) has been used for over 1000 years and consists of multiple herbs, including Radix Rehmanniae (Shu dihuang), *Cornus officinalis* (Shan zhuyu), Chinese yam (Shan yao), Poria cocos (Fu ling), Alisma (Ze xie), and Cortex Moutan Radicis (Mu danpi). In China, LDP is a frequent prescription in patients with DKD. The occurrence and development of DKD is mainly mediated by inflammatory reactions, with a close relationship existing between them.^[21,22]^ Some studies have shown that one of the risk factors for the microinflammatory state observed in DKD is caused by inflammatory cytokines and adhesion molecules.^[23]^ Tan et al^[24]^ has observed that LDP can reduce the secretion of inflammatory factors, monocyte chemoattractant protein-1 and intercellular adhesion molecule, downregulating the expression of monocyte chemoattractant protein-1 and intercellular adhesion molecule mRNA, and thus delaying glomerulosclerosis. Renal interstitial fibrosis is a pathological change occurring during DKD. TGF-β1 is the core factor in promoting fibrosis, and its expression level is closely related to the occurrence of DKD.^[25]^ Furthermore, an abnormal glomerular filtration barrier caused by podocyte injury plays an important role in the occurrence and development of DKD.^[26]^ Li et al^[27]^ have reported that LDP can protect the kidneys of diabetic nephropathy rats by increasing the expression of nephrin and podocin in podocytes.

Clinical studies assessed by Feng and Li^[28]^ have noted that LDP combined with metformin can reduce postprandial blood glucose and fasting blood glucose (FBG) effectively, significantly improving the clinical symptoms in patients with type 2 diabetes. Zhao et al^[29]^ observed that the Liuwei Dihuang decoction combined with calcium dobesilate capsules reduces postprandial blood glucose, FBG, and glycosylated hemoglobin during the treatment of diabetic nephropathy; additionally, total cholesterol, triglycerides, high-density lipoprotein (HDL), and low-density lipoprotein (LDL) were improved. In the treatment of early DKD, Ma^[30]^ has shown that losartan potassium combined with LDP can improve renal function, inhibit the chronic inflammatory reaction, control blood pressure, and reduce urinary protein excretion. A study by Cui and Ma^[31]^ has demonstrated that LDP can improve the 24-hour urinary protein (24h UP) and urinary protein excretion rate. Qiu^[32]^ has reported that LDP combined with metformin hydrochloride sustained-release tablets reduces the incidence of adverse reactions in patients with DKD. These clinical studies have confirmed that LDP can treat DKD, but the quality of these studies is inadequate.

To date, high-quality, evidence-based medicine confirming the clinical efficacy and safety of LDP in DKD treatment remains scarce. Therefore, we designed a randomized controlled trial to evaluate the efficacy and safety of LDP in the adjuvant treatment of DKD.

## 2 Methods and design

### 2.1 Objectives

This randomized controlled trial is aimed to evaluate the efficacy and safety of LDP in the adjuvant treatment of DKD.

### 2.2 Design

**Figure 1:**
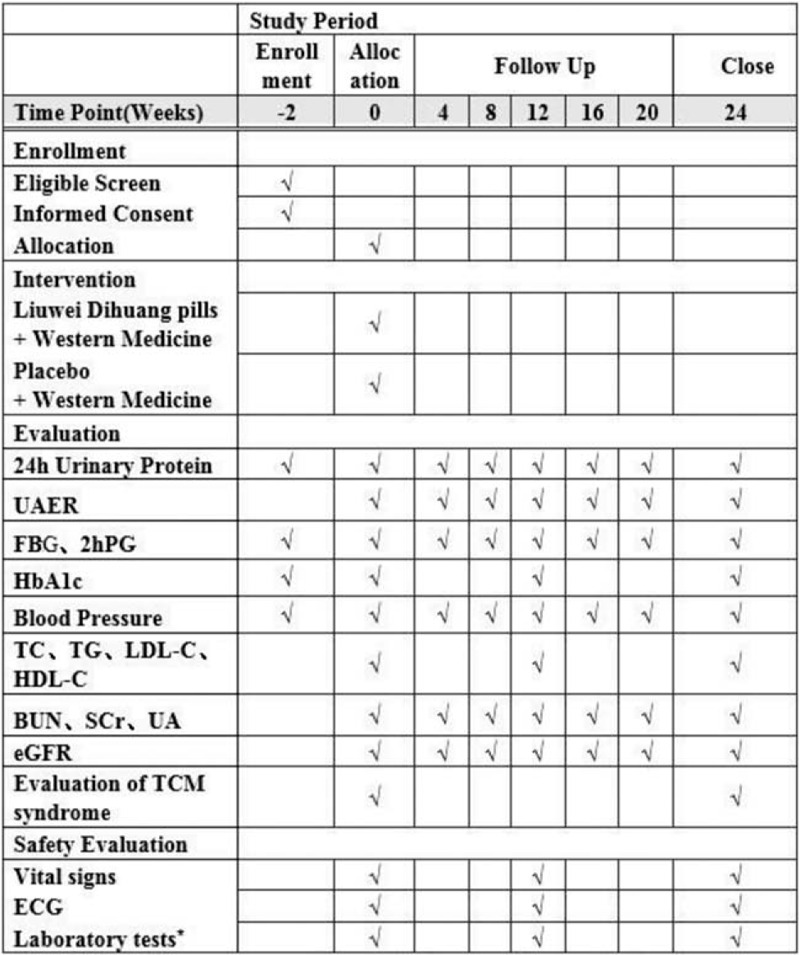
The schedule of enrollment, interventions, and assessments. BUN = blood urea nitrogen, ECG = electrocardiogram, eGFR = estimated glomerular filtration rate, FBG = fasting blood glucose, HbA1c = glycosylated hemoglobin, HDL-C = high-density lipoprotein cholesterol, 2hPG = postprandial 2-hour blood glucose, LDL-C = low-density lipoprotein cholesterol, LDP = Liuwei Dihuang pills, SCr = serum creatinine, TC = total cholesterol, TCM = traditional Chinese medicine, TG = triglyceride, UA = uric acid, UAER = urinary albumin excretion rate. ^∗^Laboratory tests include routine blood test, routine stool test, liver function test, and C-reactive protein.

**Figure 2:**
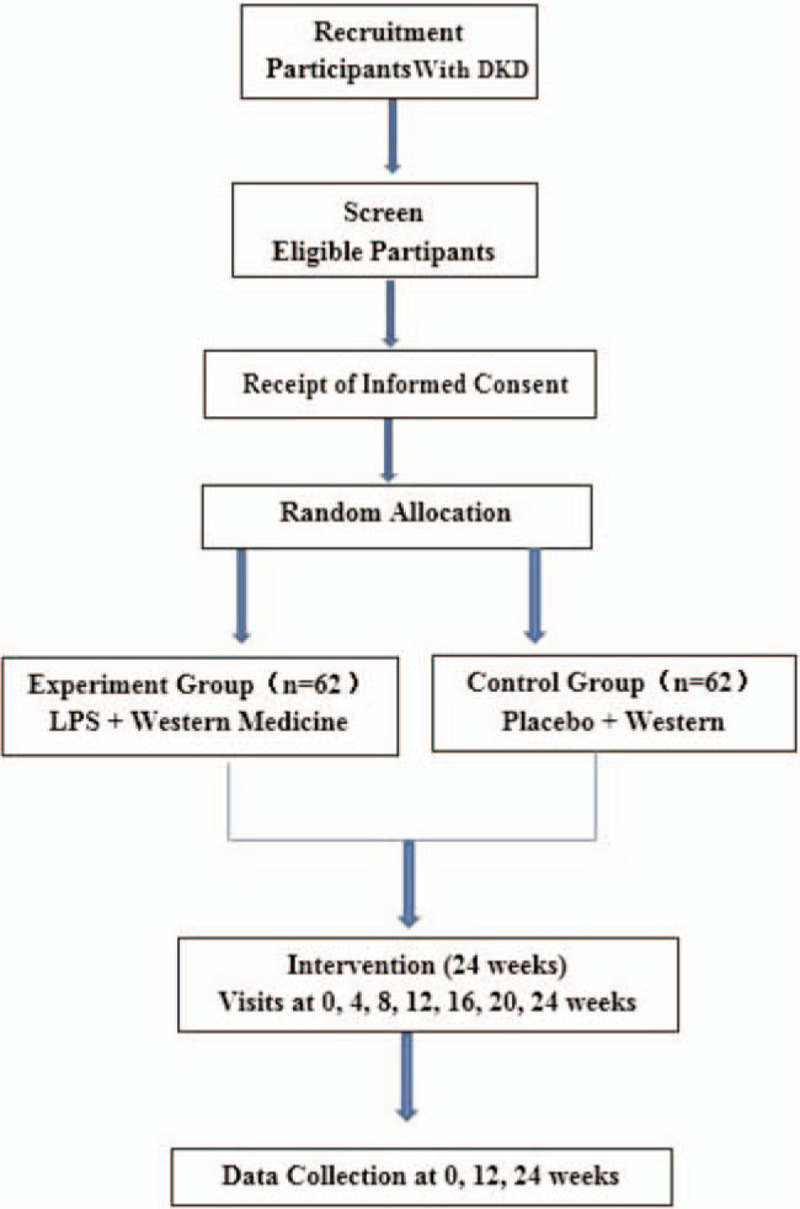
The flow chart of study design. DKD = diabetic kidney disease, LDP = Liuwei Dihuang pills.

The proposed study is a prospective, double-blind, randomized, placebo-controlled clinical trial to evaluate the efficacy and safety of LDP compared with a placebo. The clinical trial number is ChiCTR2000029800, which has been registered in the Chinese Clinical Trial Registry (http://www.chictr.org.cn/showproj.aspx?proj=10862) on February 14, 2020. A 2-week run-in period will be initiated after obtaining the written informed consent. Next, 124 eligible participants will be randomly allocated to either the LDP group or the control group in a 1:1 ratio. After allocation, patients in the experimental group will receive basic treatment and LDP, while patients in the control group will be treated with the basic treatments and placebo. Both groups will undergo a 24-week treatment, with follow-ups at 0, 4, 8, 12, 16, 20, and 24 weeks. In this study, we aim to observe the efficacy and safety of LDP when compared with western medicine alone for DKD treatment. The Standard Protocol Items: Recommendations for Interventional Trials (SPIRIT) 2013 statement will be followed by this trial protocol (see Supplemental Digital Content: Additional file 1 and Fig. 1 for the schedule of enrollment, interventions, and assessments).^[33]^ The study flow chart is shown in Figure 2.

### 2.3 Ethics approval

This study will be performed in accordance with the regulations of the Declaration of Helsinki (Edinburgh 2013 version) established by the World Medical Congress. The final amendments (version: 2.0) and the consent forms were reviewed and approved by the China Ethics Committee of Registering Clinical Trials (approval no: ChiECRCT20200028). If any amendment is made to the protocol, further approval must be obtained from the Ethics Committee.

### 2.4 Recruitment

A total of 124 participants will be recruited at the endocrine clinics of the Hospital of Chengdu University of Traditional Chinese Medicine (Chengdu, China). The recruitment will be carried out through local advertisements and doctor referrals, posters, and the Internet. The patients were included after the endocrinologist diagnoses according to the inclusion and exclusion criteria of the study. Before enrollment, participants will be provided with detailed information about the clinical study, including purpose, process, schedule, and possible risks, and benefits. All patients need to sign an informed consent form before any medical assessment.

### 2.5 Sample size

The sample size will be calculated based on the primary outcome (24h UP). According to a previous study,^[34]^ the average 24h UP level of the control group is 100.6 mg/24 h, with a standard deviation of 23 mg/24 h. After LDP treatment, the average 24 h UP level in the experimental group was 89 mg/24 h, with a standard deviation of 12 mg/24 h. The following formula is employed to calculate the sample size that allows obtaining a significantly lower 24h UP in the experimental group after 24 weeks of treatment: 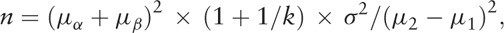


where *σ*^2^ is the overall variance, estimated as the sample variance *s*^2^: 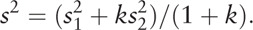


The proportion of participants between the experimental and control groups is set to 1:1 (*k* = 1). The study is designed to have a power of approximately 90% and a 2-sided significance level of 0.05 (*a* = 0.05, *β* = 0.1). *μ*_2_, *μ*_1_, *s*_1_, and *s*_2_ are the mean and standard deviations in the control and experimental groups, respectively. This resulted in *n* = (1.96 + 1.28)^2^ × (23^2^ + 12^2^)/(100.6–89)^2^ ≈ 56. Thus, after assuming a dropout rate of 10%, the sample size for patients with DKD in each group was determined as 62, and the total sample size is 124.

### 2.6 Randomization and allocation concealment

A statistician, a member of the Sichuan Traditional Chinese Medicine evidence-based Medicine Center, will generate 124 random serial numbers using the SAS 9.2 software (SAS, Cary, NC). Randomization will be accomplished after screening and baseline assessment, and eligible participants with DKD will be allocated to either the experimental group or the control group in a 1:1 ratio. The group numbers will be sequential and will be placed in sealed envelopes composed of carbon-free paper. The envelopes will be maintained by a study administrator who will not directly participate in the recruitment or follow-up of any participant, and the group numbers will be subsequently disclosed. On the day of inclusion, the administrator will open the envelope and provide the participant with their group number. Thus, all relevant individuals will remain unaware of group allocation until the study completion.

### 2.7 Double-blind treatment

This study is a double-blind design, in which neither the researchers nor the participants will be aware of the treatment group during the trial period. Both LDP granules and placebo granules will be produced, packaged, and marked by Sichuan Green Pharmaceutical Technology Development Co Ltd to ensure that the granules are identical in relevant physical characteristics, such as appearance, shape, smell, and specifications. Additionally, the research team members will not communicate with the subjects regarding treatment group allocation. In the case of emergencies only, such as serious adverse events, or if the patient requires emergency treatment, the researchers will report to the principal researcher to determine whether the treatment should be disclosed.

### 2.8 Diagnostic criteria

Participants must meet the western medicine diagnostic criteria for DKD (Table 1) as stated in the 2014 “Consensus of Experts on Prevention and Treatment of DKD” of the Chinese Diabetes Society,^[35]^ as well as the TCM syndrome diagnostic criteria of a syndrome of deficiency of kidney yin (Table 2). Syndrome differentiation will be independently determined by 2 designated associate chief or chief physicians, ranked above TCM physicians.^[36]^

**Table 1:**
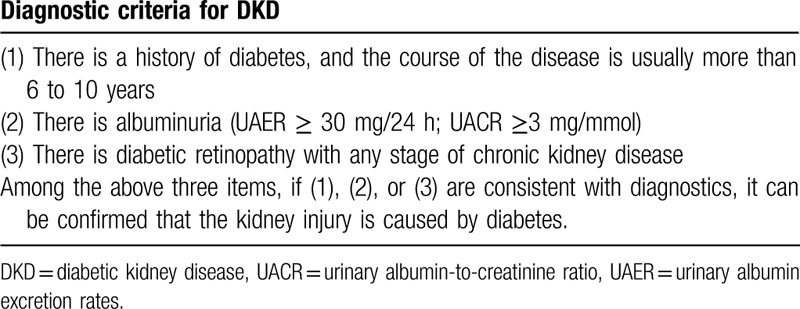
Western medicine diagnostic criteria for DKD.

**Table 2:**
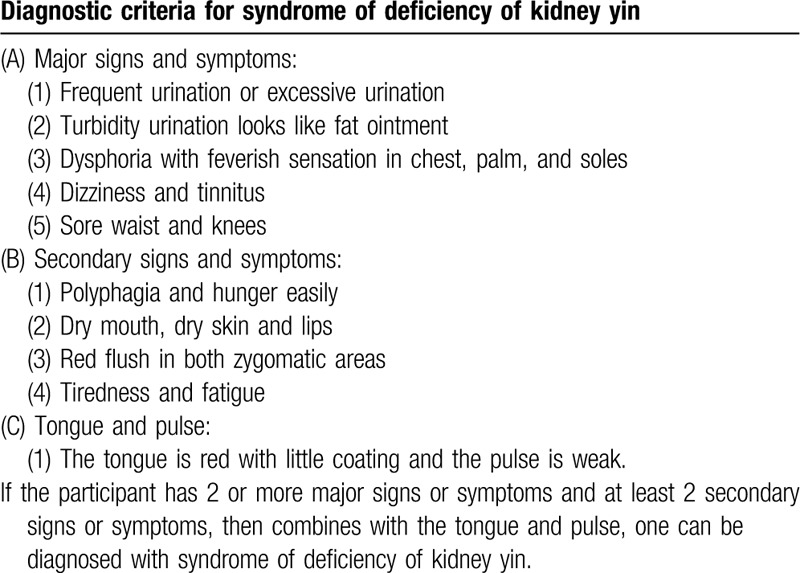
Diagnostic criteria for traditional Chinese medicine differentiation of syndrome of deficiency of kidney yin.

### 2.9 Eligibility criteria

Inclusion criteria:

1. Patients were diagnosed with DKD meeting the criteria of the 2014 “Consensus of Experts on Prevention and Treatment of DKD” of the Chinese Diabetes Society;
2. Patients aged between 25 and 75 years;
3. Estimated GFR (eGFR) ≥ 30 mL/min/1.73 m^2^ without dialytic treatments;
4. For patients with hypertension, blood pressure ≤140/90 mm Hg; for elderly, systolic blood pressure ≤150 mm Hg;
5. Glycosylated hemoglobin (HbA1c) ≤7%; for patients with serious complications such as recurrent hypoglycemia, severe microvascular or macrovascular complications, or if it is difficult to meet the standard requirements despite adequate treatment, then HbA1c ≤8%;
6. Patients with the ability to read, understand, and write research-related materials, and voluntarily comply with all test requirements;
7. Patients voluntarily participating in the study and provide written informed consent.

Exclusion criteria:

1. Severe infection, anemia, electrolyte imbalance, or acute complications of diabetes mellitus in the previous 4 weeks;
2. Severe cardiac, cerebral, hepatic, or hemorrhagic diseases, including cerebral infarction, cerebral hemorrhage, transient ischemic attack, myocardial infarction, unstable angina, heart failure, and hepatic inadequacy with aspartate transaminase or alanine aminotransferase levels more than twice the normal upper limit;
3. Use of corticosteroids or immunosuppressants in the previous 3 months;
4. Oliguria, anuria, severe edema, massive pleural, or peritoneal effusion;
5. Renal transplantation;
6. Mental disorders;
7. Pregnancy or lactation;
8. Allergy to trial drugs;
9. Participation in other clinical studies;
10. Incomplete understanding of the study, refusal to participate, or missing signed informed consent.

### 2.10 Termination and withdrawal criteria

All participants will be informed that they have the right to withdraw from the trial and that they will receive the standardized treatment if they chose to withdraw. The reason for withdrawal will be recorded precisely in the case report form (CRF). The criteria for discontinuing treatment and withdrawing patients from the research project are as follows:

1. Manifestation of diseases or conditions mentioned in the exclusion criteria during the study period;
2. Severe complications and/or general health deterioration;
3. Detection of serious adverse events;
4. Rapidly increasing serum creatinine (SCr) levels (>50% of baseline) or end-stage renal disease;
5. Violation of study protocol;
6. Voluntary withdrawal from the trial or loss to follow-up.

### 2.11 Study drugs

Study drugs to be evaluated include LDP and LDP simulation agents (placebo), which will be manufactured by the Sichuan Green Pharmaceutical Technology Development Co Ltd (Sichuan, China). The ingredients present in LDP are as follows: Radix Rehmanniae (Shu dihuang) at a dose of 24 g, *C officinalis* (Shan zhuyu) at a dose of 12 g, Chinese yam (Shan yao) at a dose of 12 g, Poria cocos (Fu ling) at a dose of 9 g, Alisma (Ze xie) at a dose of 9 g, and Cortex Moutan Radicis (Mu danpi) at a dose of 9 g. All ingredients present in the prescription will be processed to form granules by pharmaceutical manufacturers. The granules will be packaged into paper packets at a dose of 10 g. The placebos will be composed of starch, without the active ingredients. By adding various materials, the placebos will be produced such that they are identical in appearance and taste to the LDP granules.

## 3 Interventions

### 3.1 Treatment plan

Both groups will be treated using western medicine in accordance with the 2014 “Consensus of Experts on Prevention and Treatment of DKD” of the Chinese Diabetes Society.^[35]^ This includes blood glucose control (metformin hydrochloride sustained-release tablets; Trade name: Meperidine, Specification: 0.5 g × 30 seconds, Manufacturer: SPH Sine Pharmaceutical Laboratories Co, Ltd, Batch number: H20050699; Oral, 0.5 g per time, once a day) and blood pressure control (Irbesartan tablets; Trade name: APROVEL, Specification: 150 mg × 7 tablets, Manufacturer: Hangzhou Sanofi Pharmaceutical Co, Ltd, Batch number: J20080061; Oral, 150 mg once a day). Simultaneously, participants will be informed regarding diabetes health, diet control, including limited protein intake, and the need for regular exercise.

Experimental group: Patients in the experimental group will receive LDP granules at a dose of 10 g twice daily for 24 weeks, after breakfast and dinner, dissolving 10 g granules into 100 mL warm boiled water.

Control group: Participants in the control group will receive placebo granules at a dose of 10 g twice daily for 24 weeks. The measurements will be the same as in the experimental group.

During the trial period, no other drugs that could affect urinary protein or renal function will be permitted.

### 3.2 Outcome measures

Primary outcome: The primary outcome is determined as the change in 24h UP, from the baseline to the end of the treatment phase (week 24).

Secondary outcomes:

1. Changes in SCr and eGFR: eGFR will be used to assess the kidney function of participants with CKD-EPI creatinine equation of 2009^[37]^ (at weeks 4, 8, 12, 16, 20, 24).
2. Change in the urinary albumin excretion rate from baseline to week 24 (at weeks 4, 8, 12, 16, 20, 24).
3. Improvement of TCM syndromes and symptoms from baseline to week 24 (at weeks 4, 8, 12, 16, 20, 24).
4. Changes in FBG and postprandial 2-hour blood glucose (2h PG) from baseline to week 24 (at weeks 4, 8, 12, 16, 20, 24).
5. Changes in blood lipids from baseline to week 12 and week 24, including total cholesterol, triglyceride, HDL cholesterol, and LDL cholesterol (at weeks 4, 8, 12, 16, 20, 24).

### 3.3 Safety assessment

In China, LDP granules have been used for over 1000 years, and the dosage to be used in this study is within the recommended range based on the People's Republic of China Pharmacopeia (2015 edition). Moreover, we will employ a series of measures, including subjective descriptions and laboratory tests, especially focusing on heart, liver, and kidney damage, to assess the safety of LDP granules, from the time of enrollment through the follow-up period.

### 3.4 Compliance

Once patients are randomized, researchers at the study sites will make all reasonable efforts to follow the patient for the duration of the study. During each visit, adherence to the intervention will be monitored and participants will be asked to return all study containers with any unused packs of granules, including all empty containers. All examination and transportation costs will be covered, and the results of physical examinations will be explained at every visit. Before every visit, messages will be sent via WeChat or by phone to remind patients regarding the imminent data collection. Additionally, ongoing support, such as free registration and treatment advice, will be provided to the participants during the follow-up phase.

### 3.5 Adverse events

Any adverse events will be recorded in CRFs, irrespective of their relationship to the study intervention. In the case of any serious adverse events, the intervention will be immediately discontinued and a detailed description of the time, severity, relationship with the drug, and the measures taken based on standard operating procedures of the China Food and Drug Administration will be recorded. Additionally, serious adverse events will be reported to the Steering Committee and Ethics Committee within 24 hours.

### 3.6 Data management and quality control

All records will be collected in CRFs, which will be completed by a trained and qualified investigator. Once a CRF is completed, the original record will not be altered if any corrections are to be made. The completed CRFs will be reviewed by a clinical inspector.

Data entry and management will be guided by medical statisticians. To ensure data accuracy, 2 data administrators will input and proofread the data independently. After reviewing and confirming that the established database is accurate, the data will be locked by the main researchers and statistical analysts. Thereafter, the locked data or files will not be altered and will be submitted for statistical analysis by the research group. The Sichuan TCM evidence-based Medicine Center (Chengdu, China), which has no competing interests in terms of this study, will be responsible for monitoring the data. Furthermore, data audits will be performed during the trial by the Department of Science Research, at the hospital of the Chengdu University of TCM, independent of the investigators.

### 3.7 Statistical analysis

All data analyses will be conducted according to the intention-to-treat principle. Missing values will be replaced by the last observation carried forward method. Two similar participants with complete data will be double-checked to ensure that the data are correct before analysis.

The data will be analyzed using the Statistical Package for the Social Sciences version 22.0 (SPSS 22.0, Chicago, IL). The analytical methods will be selected according to the distribution characteristics of the data. The measurement data will be examined using group *t* tests or nonparametric tests, the count data will be tested using the Chi-square test or Fisher exact probability method, and the grade data will be evaluated using nonparametric tests. Compared with the baseline values, the measurement data will be assessed using paired *t* tests or nonparametric tests, and the count data will be examined using a nonparametric test. All statistical tests will be bilateral tests, and *P* values <.05 will indicate statistical significance.

## 4 Discussion

As a valuable medical treatment, TCM is currently benefiting several individuals. The essence of TCM lies in treatment based on the differentiation of disease syndromes. Currently, TCM doctors regard TCM as a basic or complementary therapy in patients with endocrine diseases. LDP is usually prescribed for patients with DKD and could relieve the symptoms of DKD. This trial aims to examine the efficacy and safety of DKD compared with a placebo in the treatment of DKD. The quality of randomized controlled trials concerning Chinese medicine presents several challenges, including faulty study design and methodology and lack of trained investigators. To ensure the quality of this study and achieve a reliable conclusion, the experimental design and study performance will be performed under strict quality control. The findings of this trial could enable alternative treatment in patients with DKD; additionally, the findings may provide scientific evidence for the use in DKD to improve patients’ symptoms and control 24h UP levels. However, the small sample size in this study is a limitation. Nevertheless, the results will provide novel evidence regarding the effectiveness of adjuvant TCM in patients with DKD.

## Acknowledgments

The authors are grateful to the Sichuan Science and Technology Program and National Natural Science Foundation of China for funding this study. They also thank Editage (www.editage.cn) for English language editing.

## Author contributions

**Conceptualization:** Qun Huang, Chunguang Xie.

**Investigation:** Keling Chen.

**Supervision:** Chuantao Zhang, Shiyun Tang.

**Writing – original draft:** Wenfan Gan.

**Writing – review & editing:** Tingting Liao, Keni Zhao.

## Supplementary Material

Supplemental Digital Content
